# Natural Sources, Pharmacokinetics, Biological Activities and Health Benefits of Hydroxycinnamic Acids and Their Metabolites

**DOI:** 10.3390/nu12082190

**Published:** 2020-07-23

**Authors:** Matej Sova, Luciano Saso

**Affiliations:** 1 Faculty of Pharmacy, University of Ljubljana, Aškerčeva 7, 1000 Ljubljana, Slovenia; 2 Department of Physiology and Pharmacology "Vittorio Erspamer", Sapienza University of Rome, Piazzale Aldo Moro 5, 00185 Rome, Italy; luciano.saso@uniroma1.it

**Keywords:** diet, natural compounds, phenolic acids, hydroxycinnamic acids, metabolites, pharmacokinetic properties, biological activities, health effects

## Abstract

Hydroxycinnamic acids (HCAs) are important natural phenolic compounds present in high concentrations in fruits, vegetables, cereals, coffee, tea and wine. Many health beneficial effects have been acknowledged in food products rich in HCAs; however, food processing, dietary intake, bioaccessibility and pharmacokinetics have a high impact on HCAs to reach the target tissue in order to exert their biological activities. In particular, metabolism is of high importance since HCAs’ metabolites could either lose the activity or be even more potent compared to the parent compounds. In this review, natural sources and pharmacokinetic properties of HCAs and their esters are presented and discussed. The main focus is on their metabolism along with biological activities and health benefits. Special emphasis is given on specific effects of HCAs’ metabolites in comparison with their parent compounds.

## 1. Introduction

**Figure 1:**
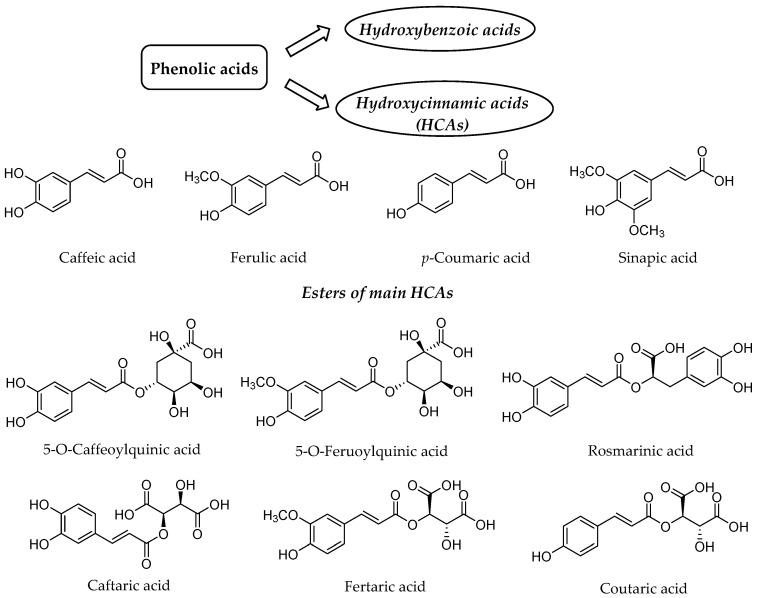
Structure of the main hydroxycinnamic acids (HCAs) and their esters as one of the major class of phenolic acids.

Our diet rich in plant food contains several health-beneficial ingredients. Among such ingredients, polyphenols represent one of the most important natural compounds. Phenolic compounds are members of probably the largest group of plant secondary metabolites and have the main function to protect the plants against ultraviolet radiation or invasion by pathogens [1,2]. They can be divided into four distinct classes based on the number of phenol rings and structural fragments connecting them, namely phenolic acids, flavonoids, stilbenes and lignans [2]. The first class generally involves the phenolic compounds possessing a carboxylic acid as the main functional group [3], thus being named as phenolic acids, which are further split into two groups, namely hydroxybenzoic and hydroxycinnamic acids (HCAs) (Figure 1). Hydroxybenzoic acids are important bioactive ingredients of edible plants [4,5,6]; however, more common and studied phenolic acids are HCAs, which are present in the variety of plant-based foods, especially in fruit, vegetables and seeds [3]. HCAs possess phenylpropanoid C6-C3 structure as the main chemical scaffold and are recognized by the presence of hydroxyl group(s) on the aromatic ring(s) and a carboxyl group in the lateral chain [7,8]. The number and position of hydroxyl groups and other substituents contribute to the diversity of HCAs. The most abundant HCAs in nature are *para*-coumaric, caffeic, ferulic, and sinapic acids (Figure 1) [8,9]. In nature, all four acids are rarely present in a free form and are usually esterified with quinic and tartaric acids or various derivatives of carbohydrates [10]. Chlorogenic acids are one the most abundant esters including the whole set of HCAs esters with quinic acid, namely caffeoyl-, feruloyl-, dicaffeoyl- and coumaroylquinic acids [11,12]. The most common representative is 5-*O*-caffeoylquinic acid (Figure 1) often referred to as chlorogenic acid [12]. An ester of caffeic acid and 3,4-dihydroxyphenyllactic acid is called rosmarinic acid (Figure 1), which is one of the most abundant caffeic acid ester in the plant kingdom besides chlorogenic acids [13].

Caffeic acid presents up to 70% of whole HCAs in fruits, whereas ferulic acid is the prevalent HCA in cereal grains [10]. The daily consumption of HCAs varies significantly between individuals [14,15,16], which is attributed not only to different intake but also diverse metabolism and absorption from the gut. The bioavailability and metabolism of HCAs and their conjugates is thus of high importance for health benefits for particular individual.

Herein, we will briefly present natural sources and pharmacokinetic properties of HCAs and their esters. Afterwards, the main focus will be on their metabolism, biological activities and health benefits with emphasis on specific effects of HCAs mediated by their metabolites.

## 2. Dietary Intake and Nutritional Importance of HCAs

HCAs are one of the most widely distributed naturally occurring phenolic acids being typically present in the form of esters with quinic, shikimic or tartaric acid, saccharides, flavonoids or with plant structural elements (i.e., cellulose, lignin and proteins) [12,17,18]. HCAs are considered as important constituents of our diet, contributing to taste, color, nutritional value and health benefits [14]. HCAs are thus present at a wide concentration range in our everyday food and drinks, including fruits (apples, berries, plums, cherries, peaches and some citrus fruits), vegetables (carrots, salad, cabbage, eggplant, and artichoke), cereals, beverages (tea, coffee), grapes and wine [14,19,20,21,22]. HCA derivatives represent about 18% of all phenolic compounds in apples with chlorogenic acid as the most abundant HCA in the entire apple (up to 87% of the total HCA amount) [23], whereas *p*-coumaric, caffeic and ferulic acids are encountered in blueberry fruits [22]. Indeed, caffeic acid is the most abundant in fruits (between 75 and 100% of the total HCA content) with the highest quantities in the range of 0.5 to 2 g in blueberries, kiwis, plums, cherries, and apples, whereas ferulic acid is ubiquitous in cereal grains, which represent its major dietary source [24]. For example, ferulic acid is the prevalent phenolic acid in barley brans and seeds [22] and is also present in blueberries and blackberries ranging from 2.99 to 16.97 mg/g fresh weight [25]. Similarly, the levels of *p*-coumaric and caffeic acids in blueberry fruits varies from 0.40 to 15.78 and 1.38 to 6.32 mg/g fresh weight, respectively [25]. The most abundant HCAs in cranberry fruit are *p*-coumaric and sinapic acids with approximately 0.25 and 0.21 g/kg fresh weight, respectively [26]. All major HCAs are also present in numerous vegetables, with an average amount of total phenolic acids up to 32.0 mg/100 g fresh weight [27]. The major soluble HCAs identified in breeding vegetables are chlorogenic acids (eggplant, carrot, basil, spinach, Chinese cabbage, parsnip, lettuce, pepper, cauliflower, turnip, green bean, tomato), *p*-coumaric acid (radish, pepper, cauliflower, white cabbage, onion, zucchini, cucumber), ferulic acid (red beet, radish, pepper, turnip, cucumber), caffeic acid (carrot, broccoli, zuccini) and sinapic acid (broccoli, Chinese cabbage, cauliflower, turnip, white cabbage, pea) [27]. In another study, ferulic acid and caffeic acid were identified at high concentrations in spinach (18.0–41.4 mg/kg dry weight) and garlic (1.7–28.3 mg/kg dry weight), respectively, while chlorogenic acid was determined as the most abundant HCAs in artichoke (37.8–734.7 mg/kg dry weight) [28]. Chlorogenic acids are also one of the main constituents of green coffee beans [29], with daily intake in the range from 120 to 594 mg for regular coffee drinkers [30], whereas caffeoyl- and *p*-coumaroyl-quinic acids were identified in tea leaves [31]. Indeed, coffee beans are one of the richest sources of chlorogenic acids in the diet, leading to highly variable levels in coffee brews according to the literature data [30]; however, typical values of chlorogenic acids and their main lactones are in the range from 50 to 200 mg/mL. Ferulic, *p*-coumaric and caffeic acid are located in skin’s vacuoles and pulp cells of grapes being esterified with tartaric acid and named as fertaric, *p*-coutaric and caftaric acids (Figure 1), respectively [32]. Caftaric acid thus presents an important phenolic compound in white (6–73 mg/L) or red wine (46–141 mg/L) [14,19,33]. HCAs can be also found in mushrooms. Small amounts of caffeic, ferulic and *p*-coumaric acids were determined in the extracts from Polish wild growing edible mushrooms with the exception of *Pholiota mutabilis*, which contained 29.10 mg/kg dry weight of *p*-coumaric acid [34].

HCAs are generally ingested daily in high amounts, which vary significantly between individuals—the estimated intake of 46.3 to 78.9 mg/day for children and 153.6 to 231.8 for adults was determined in cross-sectional analysis of UK National Diet and Nutrition Survey Rolling Programme [15]. Another study estimated the average phenolic acid consumption for men and women of 222 mg per day, dominated by caffeic acid with 206 mg of daily intake [16]. The prevailing dietary sources of HCAs are coffee and fruits with 92% of the caffeic acid and 59% of the *p*-coumaric acid intake, respectively [16].

In addition to taste, color and nutritional value, HCAs are of high importance due to their health-beneficial effects [3,14,35], which are described in details in the Section 4.

## 3. Bioavailability of HCAs

From the nutritional point of view, bioavailability is described as the fraction of a given food which our body can utilize and it is highly affected by various factors, such as bioaccessibility, the food matrix effect, transporters, molecular structures, metabolizing enzymes and absorption [36].

### 3.1. Food Processing and Bioaccessibility

In order to exert a range of health beneficial effects after the consumption of plant-derived food, bioactive phytochemicals need to withstand food processing and release from the food matrix after ingestion [36,37]. Furthermore, after release in the gastrointestinal tract (GIT) (bioaccessibility), the uptake of the active compounds along with metabolism in the GIT and liver is also highly important to reach the target tissue responsible for their biological activity. 

The bioavailability of phenolic acids rely upon their form (free or conjugated) present in the food matrix; thus, it could also be affected by food processing [14]. The main example of high food processing influence is cereals, where the majority of the edible fiber-bound phenolic acids are esterified to the cell walls and are common components of complex structures (such as hydrolysable tannins, lignins, organic acids), thus being poorly bioavailable [14,38]. For example, ferulic acid as a dominant HCA in oats is bound to the cell wall arabinoxylan or dimerized through oxidative cross-linking [38]. Many conventional processing techniques (i.e., cleaning and heat treatment, dehulling and cutting, flaking or milling, germination) remove HCAs or increase the levels of free acids in oat and cereal food products [38]. Therefore, an optimized processing has a noteworthy influence on the absorption of bioactive compounds such as HCAs, being especially important in case of tightly-bound ferulic acid [39].

The second important factor affecting the bioavailability of HCAs after appropriate food processing is the release from the food matrix after ingestion, which can be defined by the term bioaccessibility. Bioaccessibility is affected by the composition of the consumed food matrix and physicochemical properties (e.g., pH, temperature and the texture of the matrix) [36]. A considerable percentage of HCAs exhibit low bioaccessibility because of the structural complexity of the plant’s cell wall [40]. The investigation of the effects of boiling and extrusion processes applied in sorghum bran, a known source of HCAs, showed the improvement of the HCAs’ release and rise of the antioxidant capacity. In case of ferulic acid from boiled or extruded sorghum bran, higher bioaccessibility in the GIT was observed. Therefore, it was showed that food matrix and in vitro digestion conditions along with applied technological processes have an important impact on the release of HCAs [40]. Furthermore, the interaction with digestive enzymes could also alter the bioaccessibility of HCAs.

### 3.2. Absorption, Distribution, Metabolism and Excretion of HCAs

The rate and extent of the absorption of HCAs from the GIT to the systemic circulation generally depends on their structure [41]. It is known that the presence of an ester moiety results in lower HCAs absorption [42]. Several studies demonstrated that bound HCAs (for instance ferulic and caffeic acid esters) have reduced absorption capacity through enterocytes in the gastrointestinal wall compared to their free forms [42,43]. On the other hand, HCAs in a free form are rapidly absorbed throughout the GIT, whereas HCA esters or HCAs attached to cell walls are hydrolyzed by esterases before absorption [42]. According to in situ or ex vivo absorption models, ferulic, caffeic and *p*-coumaric acids could be absorbed from the stomach, jejunum, ileum and colon based on the studies in rats, which were summarized in a brief review by Zhao and Moghadasian [44]. In case of ferulic acid, the colon represents the key site of absorption due to the presence of microbial cinnamoyl esterases, which facilitate its release from the food matrix or parent compounds [45,46]. In another study using the in vitro model for the colonic epithelium (Caco-2/HT29-MTX co-culture cell model), it was suggested that ferulic acid is absorbed via two distinct mechanisms, i.e., passive transcellular diffusion and facilitated transport [46]. Furthermore, the active absorption via the monocarboxylic acid transporter was proposed for some HCAs (i.e., ferulic acid [47], *p*-coumaric acid [48]) in Caco-2 cells. On the other hand, caffeic acid has low affinity for this transporter and is generally more efficiently absorbed via paracellular pathways, i.e., paracellular diffusion [49]. It was shown that the absorption efficiency of HCAs in vivo is increased in the order from rosmarinic acid, caffeic acid to *p*-coumaric acid [50]. The absorption of caffeic acid has also been investigated in many other studies [43,51,52,53]. The in situ vascularly perfused rat intestinal preparation, which enables precise and indirect assessment of the contribution of intestinal absorption, was employed to determine the extent of caffeic acid absorption of 12.4% after intraduodenal administration. Furthermore, poor permeability across the Caco-2 cell monolayer was shown for caffeic acid [53]. Thus, it was proposed that the poor bioavailability of caffeic acid in rats (determined as 14.7% in this study) is connected to low absorption from the GIT along with low permeability across the Caco-2 cell monolayer. 

One of the most abundant sources of caffeic acid in nature is 5-O-caffeoylquinic acid, which is most likely hydrolyzed to caffeic and quinic acids by esterases from colonic microflora [54] and are not degraded and absorbed in the upper GIT [45]. However, the study from Olthof and coauthors showed that about 33% of chlorogenic acid and 95% of caffeic acid is absorbed in the small intestine of humans [51]. The increased hydrophilic characteristics of the quinic moiety in ester most likely have an impact on the rate and extent of absorption and are responsible for changing its permeability across the epithelium [42]. Thus, part of ingested 5-O-caffeoylquinic acid will reach the blood circulation, while most of it will proceed to the colon and hydrolyse to caffeic and quinic acids. On the other hand, *p*-coumaric acid exhibits higher bioavailability compared to chlorogenic, caffeic and ferulic acids, being absorbed in rats throughout the whole GIT (including stomach, jejunum, ileum and colon) having the highest absorption rate in jejunum [44,55]. While *p*-coumaric acid in a free form is easily and quickly absorbed in the upper GIT, its conjugates exhibit much fewer and slower absorption, with higher proportion reaching the colon [55].

The distribution of HCAs within the body along with high absorption has a really important influence on health-beneficial effects of HCAs [50]; however, there have not been many studies conducted about the distribution of HCA to target tissues. In a pharmacokinetic study of caffeic acid from the methanol seed extract of *S. cumini* in rats, its disposition from the plasma to more perfused tissues was observed in one hour after absorption [56]. Even though caffeic acid was rapidly absorbed from the GIT of rats, only small amounts (19.1%) of ingested dose reached the circulatory system. According to the data obtained about clearance (21.86h) and volume of distribution (4.378), a good safety profile for caffeic acid due to a small time of exposure was proposed [56]. Another study in ddY mice demonstrated distribution of caffeic acid in the plasma, liver and skin following oral administration, absorption and metabolism into conjugated and/or methylated derivatives [57]. It was showed that caffeic acid is efficiently transported in the skin, being able to prevent the damage by UVA-induced generation of reactive oxygen species. The pharmacokinetic study of ferulic acid as the main metabolite of angoroside C in rats showed that ferulic acid is also distributed in some major organs, namely liver, lung, spleen and kidney, with the highest concentration detected after 6 hours, especially in kidneys [58]. In order to follow the distribution of polyphenolic compounds including major HCAs (caffeic, ferulic, sinapic and *o*-, *m*- and *p*-coumaric acids) to target tissues in rats the intravenous administration of 23 polyphenol microbial metabolites was performed by Gasperotti et al. [59]. The kinetics of distribution of the aforementioned HCAs and metabolites in the blood, brain, heart, liver, kidney, and urine showed their accumulation in the kidneys. Due to their low concentrations in liver, it can be concluded that absorbed HCAs’ metabolites from the colon are subjected to limited first-pass metabolism leading to rapid distribution to the other organs after absorption [59]. The pharmacokinetic study of 5-O-caffeoylquinic acid in rats demonstrated distribution to the highly perfused tissues (e.g., liver, kidneys), indicating the importance of an organ’s blood flow and perfusion rate for distribution process of 5-O-caffeoylquinic acid [60]. It was also found in the lung, heart, and spleen; however, the highest amounts were present in liver. Following the levels of 5-O-caffeoylquinic acid in organs indicated its rapid metabolism since it could not be detected there anymore after 4h [60]. In another study in rats, 5-O-caffeoylquinic acid was quickly absorbed after its intranasal administration and high levels in cerebrospinal fluid of rat brain was observed indicating direct nose-to-brain distribution of 5-O-caffeoylquinic acid, which implies the potential use in the therapy of neurodegenerative disorders [61].

**Figure 2:**
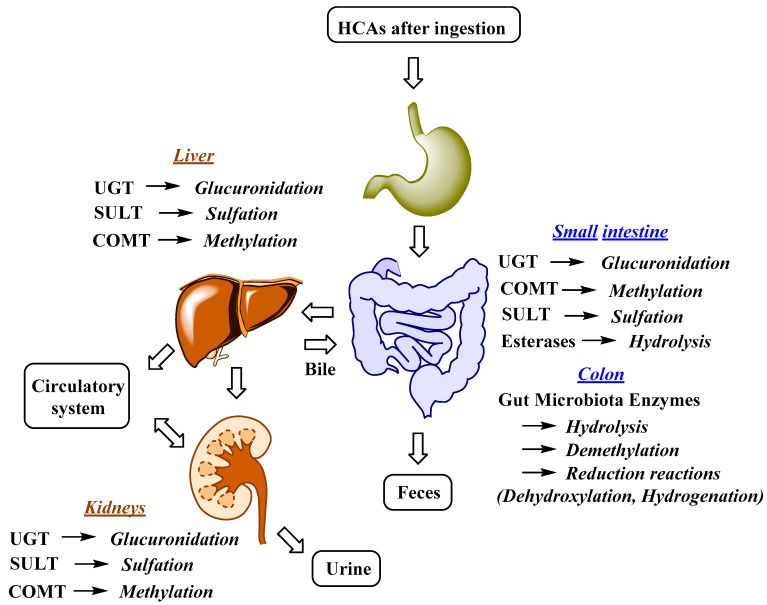
Major metabolic reactions, enzymes and organs involved in HCAs metabolism; UGT, uridine-5′-diphosphate-glucuronosyltransferase; COMT, catechol-O-methyltransferase; SULT, sulfotransferase [62,63].

The uptake and distribution of HCAs is also highly dependent on their metabolism, which can occur in the GIT, liver and kidneys (Figure 2) [62,63]. Indeed, first-pass metabolism has an important impact on the bioavailability, and consequently also on the bioefficacy of HCAs. Following the ingestion and absorption, HCAs are conjugated by glucuronidation, methylation, and sulfation reactions, which are catalyzed and regulated by specific enzymes (Figure 2) [63]. The conjugation of the hydroxyl group(s) of phenolic compounds, which is/are present in HCAs, can occur with glucuronate or sulfate [64]. The glucuronidation and sulfation of HCAs in the GIT and liver are catalyzed by uridine-5′-diphosphate-glucuronosyltransferases (UGTs) and sulfotransferases (SULTs) [65]. Furthermore, O-methylation also occurs and is catalyzed by catechol-O-methyltransferases (COMTs). HCA esters (e.g., chlorogenic acids) are hydrolyzed by esterases [66]. According to in vitro studies, chlorogenic acids display different susceptibility to hydrolysis, which can occur in the stomach or upper GIT, with 5-caffeoylquinic acid being hydrolyzed more readily by intestinal chlorogenate esterase compared to 3- and 4-caffeoylquinic acid [67]. Glucuronidation, sulfation, methylation and also hydrogenation can take place in enterocytes and liver, whereas conjugation with glycine is acknowledged to only kidneys and liver. In the latter demethylation and dehydrogenation also occurs [68]. The intestinal metabolism is also highly affected by gut microbiota (Figure 2). The human GIT microbiota transform the ingested HCAs to metabolites that usually show higher activity and better absorption compared to the parent compounds. Gut microbiota metabolic transformations can be divided into three main reactions: hydrolysis (O-deglycosylations, hydrolysis of esters), cleavage (C-ring cleavage, demethylation) and reductions (dehydroxylation and hydrogenation) (Figure 2) [62].

**Figure 3:**
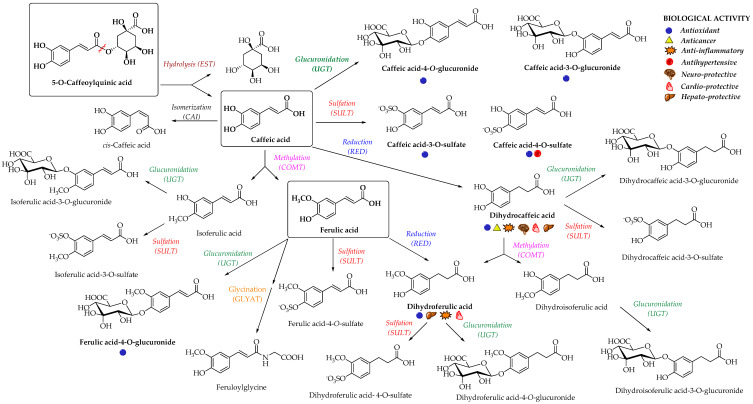
Proposed metabolic pathways and metabolites of 5-O-caffeoylquinic, caffeic and ferulic acids; EST, esterase; CAI, caffeic acid isomerase; RED, reductase; COMT, catechol-*O*-methyltransferase; SULT, sulfotransferase; UGT, uridine-5′-diphosphate-glucuronosyltransferase; GLYAT, glycine-N-acyltransferase [52,70,71]. The main biological activities of selected metabolites are presented with appropriate symbols.

Enterocyte-like differentiated Caco-2 cells are one of the most utilized in vitro models for examination of small intestinal epithelium metabolism [69]. The investigation of the metabolism of the main HCAs and their esters in vitro in the Caco-2 model demonstrated glucuronidation, methylation, and sulfation of free and methyl-HCAs along with hydrolysis, which occurred extra- and intracellularly. According to the results obtained in this study, sulfation could be the preferential metabolic reaction for HCAs in the epithelium of small intestine [69]. In case of ferulic acid, which is efficiently transported over the intestinal barrier in a free form, only low amounts of conjugates (feruloyl-glucuronide or sulfate, as well as some free dihydroferulic acid) with feruloyl-glucuronide as a main metabolite was observed in the in vitro model for human small intestinal epithelium (Caco-2/HT29-MTX co-culture cell model) [46]. The proposed main metabolic pathways and metabolites of 5-O-caffeoylquinic, caffeic and ferulic acids are presented in Figure 3 [52,70,71].

In a study from Stalmach et al. [71], the metabolite profiling of HCA’s derivatives in human plasma and urine following the coffee ingestion was performed. Caffeoyl and feruloylquinic acids are one of the most abundant coffee ingredients; however, only trace levels of 5-caffeoylquinic acid and low levels of three different feruloylquinic acids appeared in the circulatory system. This is due to the presence of intestinal esterases [66], which hydrolyse 5-caffeoylquinic acid to caffeic acid that is further metabolized to caffeic acid-3-O-sulfate (Figure 3) [71]. As already noted, the hydrolysis of the remaining 5-caffeoylquinic acid into caffeic and quinic acids is catalyzed by esterases provided by the gut microbiota [54]. In addition to caffeic acid-3-O-sulfate, ferulic acid-4-O-sulfate (Figure 3) was also detected in plasma, probably as a result of a parallel ferulic acid metabolism involving the hydrolysis of feruloylquinic acids, caffeic acid methylation by COMTs and conversion of caffeoylquinic acids to feruloylquinic acids, thereby contributing to the ferulic acid pool [71].

The hepatic uptake and metabolism of HCAs was also studied using human hepatoma HepG2 cells as a hepatic model system [72]. Moderate uptake of caffeic and ferulic acids was observed, while chlorogenic acid showed null metabolism and very limited absorption. In case of caffeic acid, methylation was found to be the preferential metabolic pathway along with sulfation and glucuronidation, whereas ferulic acid converted to glucuronides as the only metabolites [72]. Another study in the human liver S9 homogenates showed that sulfation compared to glucuronidation is more preferred, being the most efficient and high-affinity pathway for HCAs’ metabolism in liver [65]. The highest efficiency of conjugation was demonstrated in caffeic acid, followed by ferulic, dihydrocaffeic, isoferulic and dihydroferulic acids [42,65]. Similarly, absorbed *p*-coumaric acid can also undergo the conjugation with glucuronide, sulfate and sulfoglucuronide (diconjugation with sulfate and glucuronic acid) in the liver [55]; however, sulfoglucuronide is more typical for ferulic acid and was showed as the leading metabolite (60–70% of the total) along with ferulic acid glucuronide and sulfate in the rat’s plasma following the administration of free ferulic acid or its sugar esters [73]. The bioavailability study of yerba mate containing phenolic compounds in humans led to the identification of 34 metabolites in biological fluids with sulfates of caffeic, ferulic and isoferulic acids as the prevailing metabolites [74]. The main metabolites determined in plasma as a consequence of delayed colonic absorption after colonic microbiota metabolism were reduced forms of HCAs (i.e., dihydroferulic, dihydrocaffeic and dihydroisoferulic acids) and their phase II conjugates (i.e., dihydrocaffeic acid-4-O-sulfate, dihydroferulic acid-4-O-glucuronide, dihydroisoferulic acid-3-O-glucuronide, dihydroferulic acid-4-O-sulfate and dihydroisoferulic acid-3-O-sulfate) in addition to feruloylglycine [74]. 

**Figure 4:**
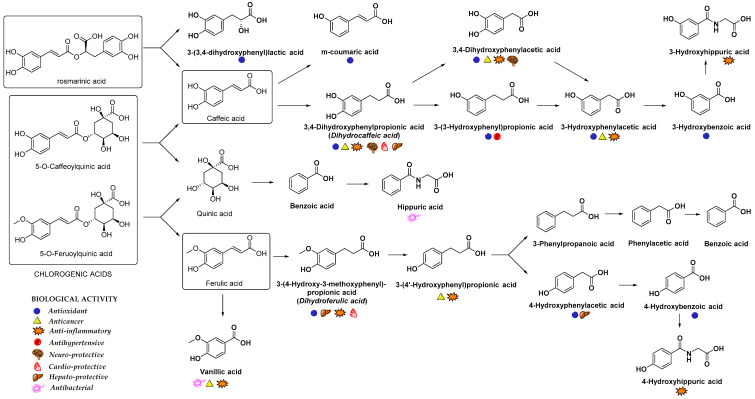
Main metabolites of rosmarinic, chlorogenic, caffeic and ferulic acids that appeared after biotransformation by gut microflora [67,75,88,89]. The main biological activities of selected metabolites are presented with appropriate symbols.

In addition to glucuronidation and/or sulfation, HCAs can also oxidize into benzoic acid derivatives that are further converted into hippuric acid derivatives [14]. For example, the metabolism of chlorogenic acid by GIT microbiota into diverse aromatic acid metabolites (e.g., *m*-coumaric acid, benzoic and phenylpropionic acids derivatives) was observed [75]. Indeed, the metabolites of microbial origin, such as *m*-coumaric, 3,4-dihydroxyphenylpropionic, 3-(3-hydroxyphenyl)propionic acid, 3-hydroxybenzoic, 3-hydroxyhippuric and hippuric acids (Figure 4) were detected in plasma and urine after chlorogenic acid diet in rats indicating the high importance of gut microflora metabolism to bioavailability of HCAs [75]. It was suggested that the preferred route of caffeic acid metabolism in rats is to 3-(3-hydroxyfenil)propionic acid, whereas 3-hydroxyhippuric acid is mainly excreted in the urine of humans [76]. Hippuric acid is mainly produced as a result of quinic acid moiety metabolism; however, it can also originate from other metabolites in the caffeic acid metabolic pathway. According to the gastrointestinal model studies, the gut microbiota metabolism of chlorogenic, ferulic and caffeic acids (Figure 4) generates various cinnamic acids (caffeic, ferulic, coumaric, dihydrocaffeic and cinnamic acids), phenyl substituted propionic acids (3-(3,4-dihydroxyphenyl)propionic, 3-phenylpropionic, and 3-(4-hydroxy-3-methoxyphenyl)propionic acids), benzoic acids (vanillic, 3-hydroxybenzoic and benzoic acids) and 3-hydroxyphenylacetic acid [77,78]. In case of *p*-coumaric acid, the observed plasma metabolites were m-dihydrocoumaric acid and dihydrocoumaric acid-O-sulfate [55]. 

Due to the fast and considerable metabolism of HCAs, the majority of their metabolites are quickly excreted in bile (largely conjugated metabolites) and urine (small conjugates, e.g., sulfates) [21,79]. There have been many studies in rats and humans about the HCAs’ excretion via the urinary and biliary pathways and they were described or reviewed elsewhere [21,51,52,76,80,81,82,83,84]; thus, we will briefly mention only the recent ones. The recent study of the urinary excretion rates of the main HCAs in non-fasted rats demonstrated the highest excretion rate for ferulic acid, followed by caffeic and *p*-coumaric acids, with all being absorbed, metabolyzed, and excreted in the urine within 6h, while chlorogenic acids showed much smaller and slower urinary excretion (up to 48h) [85]. Relatively fast urinary excretion (up to 8h) of 30 various metabolites was observed following the ingestion of oat bran in humans. The highest concentrations in the urine were determined for vanillic and hydroxylated hippuric acids (especially at positions 3 and 4), and ferulic acid-4-O-sulfate [86]. Generally, in most studies, up to date HCAs’ derivatives in the form of sulfates, glucuronides and on a smaller scale also glycine conjugates have been usually excreted in the urine [87]. Many other metabolites have also been identified in the urine resulting from metabolism in liver and kidneys or from biotransformations by gut microbiota and are summoned in a recent book by Farah [87]. 

## 4. Biological Activities and Health Benefits of HCAs and Their Metabolites

HCAs are of high importance due to their health-beneficial effects [3,14,20,21,35] and as cosmeceutical ingredients [90]. HCAs are mainly recognized as potent antioxidants [91,92], thereby being involved in the prevention of several diseases connected to oxidative stress, i.e., cardiovascular and neurodegenerative diseases, and cancer [35,93,94]. Anti-inflammatory [95] and antimicrobial activities [90,92] are also acknowledged to several HCAs and their derivatives. The biological activities of the main representative HCAs (e.g., chlorogenic acid(s), caffeic, ferulic and coumaric acids) have been already thoroughly reviewed and will be mentioned only briefly. The main focus will be on HCAs’ metabolites and their health benefits along with comparison with their respective parental HCAs.

### 4.1. Antioxidant Activity

Metabolic processes in our body and/or other external factors generate free radicals and other reactive oxygen (ROS) or nitrogen species (RNS) leading to oxidative stress, which has been associated with the pathogenesis of numerous human diseases, namely cancer, diabetes, inflammatory (arthritis, atherosclerosis, vasculitis, lupus erythematous, glomerulonephritis, adult respiratory diseases syndrome), neurodegenerative (Alzheimer’s disease, Parkinson’s disease, muscular dystrophy), autoimmune and cardiovascular diseases (heart diseases, stroke, hypertension) [96,97,98]. Antioxidants are considered as important players against the harmful effects of ROS, being engaged in the prevention of oxidative stress-related diseases [99]. Phenolic compounds including HCAs are known as potent antioxidants mainly because of their high redox properties; they could serve as hydrogen donors, singlet oxygen quenchers, and efficient reducing and metal chelating agents [98,100]. In numerous reports, potent antioxidant properties of HCAs and their derivatives are described [92,101,102,103] due to the presence of a phenolic hydroxyl group that is able to react with free radicals and other ROS to create a resonance-stabilized phenoxyl radical, and propenoic side chain that could stabilize the phenoxyl radical via conjugated double bond [92,102]. Thus, all HCAs’ metabolites possessing the free phenolic group are still able to exert antioxidant activity. The antioxidant efficacy of HCAs with monophenolic fragment is significantly increased by the addition of methoxy substituents or a second hydroxyl group in the *ortho* position which enables intramolecular hydrogen bonding [92,102,104]. Thus, it is evident that caffeic acid with two phenolic hydroxyl groups and conjugation abilities is a potent antioxidant, which has been confirmed in numerous in vitro assays for antioxidant activity determination (2,2’-azino-bis(3-ethylbenzothiazoline-6-sulfonic acid (ABTS), 2,2-diphenyl-1-picrylhydrazyl (DPPH) and ferric thiocyanate methods, superoxide anion radical scavenging activity measurement, etc.) [105]. The antioxidant properties of caffeic acid including its mode of action and in vivo activity were thoroughly reviewed a few years ago [106]. Recently, it was also demonstrated that caffeic acid as well as 5-O-caffeoylquinic acid possess the ability to scavenge the NO radical in a dose-related manner [107]. The antioxidant properties of all chlorogenic acid isomers were endorsed by chemical-, cell- and animal-based assays [108]. Ferulic and *p*-coumaric acids are also potent antioxidants, which is evident from a recent review [109] and in vitro assays as well as protective effects against oxidative stress in PC12 cell model evaluation [110], respectively. 

To examine the importance of a double bond in caffeic acid for antioxidant activity, both dihydrocaffeic and caffeic acids were tested in various assays [111]. The efficiency of DPPH and lipid peroxyl radical scavenging was similar for both acids stressing the importance of two phenolic hydroxyl groups, while dihydrocaffeic acid exhibited a less potent inhibition of the copper-induced oxidation of human low-density lipoproteins compared to caffeic acid. However, dihydrocaffeic acid on the other hand more efficiently increased the oxidative stability of lard at 60 °C [111]. This reflects the importance of surrounding milieu where oxidation occurs and shows that the fundamental antioxidant activity of caffeic acid does not rely on the presence of conjugated double bound.

Rosmarinic acid is metabolized into caffeic and 3-(3,4-dihydroxyphenyl)lactic acid and all three compounds as well as ferulic and *m*-coumaric acids were tested for their antioxidant activity in DPPH and cellular assays [88]. The radical-scavenging activity of first three compounds was similar to quercetin, while ferulic and *m*-coumaric acids were less potent or inactive, respectively. In cellular assays, all HCAs and 3-(3,4-dihydroxyphenyl)lactic acid exhibited low or no capacity to protect against the oxidative stress induced by *tert*-butylhydroperoxide (*t*-BuOOH). Additional evaluation of the methyl ester of rosmarinic acid showed similar antioxidant potency in all assays proposing that the ionization of HCAs at physiological pH leads to a considerable reduction in their intracellular accumulation and thus lowers their intrinsic antioxidant potency observed in the non-cellular assays [88].

Even though the glucuronidation and sulfation of HCAs most likely have an important impact on HCAs’ antioxidant activity because it substitutes the essential phenolic OH groups for the antioxidant properties, it was demonstrated that some of caffeic and ferulic acids’ metabolites still retain potent antioxidant activity (Table 1) with potential significant antioxidant action in vivo [112]. Ferulic acid-4-O-glucuronide and ferulic acid-4-O-sulfate showed weak antioxidant activity, whereas caffeic acid monosulfate derivatives were only 4-fold less potent antioxidants compared to the parent compound. The ferric-reducing activity of caffeic acid 3-O-glucuronide was quite high; however, it was around 50% less in comparison with caffeic acid, while it was in the same range compared to iron sulfate. Contrarily, caffeic acid 4-O-glucuronide has around 10-fold lower ferric-reducing ability compared to 3-O-glucuronide; therefore, the presence of a phenolic hydroxyl group at position 4 appears to be essential for ferric-reducing activity [112]. This suggestion was also confirmed by the measurement of antioxidant activity with the ABTS method, where caffeic acid and its 3-O-glucuronide share similar antioxidant activity, whereas caffeic acid 4-O-glucuronide has around three-fold less potent antioxidant ability. Glucuronidation could also occur at the carboxylic group, generating the so called acyl glucuronides, which retain unsubstituted hydroxyl groups and thus antioxidant properties of the parent compound [112]. Indeed, Piazzon et al. demonstrated that acyl glucuronide of ferulic acid, which is synthesized in liver, possesses potent antioxidant activity and thus present the important contribution to the plasma antioxidant potential [112].

The CuSO_4_-induced LDL autoxidation system was selected to study the antioxidant activity of ferulic acid *p*-glucuronide since the oxidative modification of LDL could present a key factor in the pathogenesis of atherosclerosis [113]. It was found that ferulic acid *p*-glucuronide with hydrophobic feruloyl and a hydrophilic sugar moieties showed more potent activity compared to ferulic acid in the LDL autoxidation system.

Dihydrocaffeic and dihydroferulic acid as the major circulating metabolites of chlorogenic acids with high t_1/2_ in the human body (detected in the urine up to 48h following the intake of one coffee [138]) were evaluated in human hepatoma HepG2 cells, which were subjected to oxidative stress elicited by *t*-BuOOH [115]. It was showed that pre-treatment with dihydrocaffeic acid hindered the cytotoxicity and macromolecular damage; furthermore, it also led to the dose-dependent recovery of reduced glutathione and elevated ROS levels, and antioxidant enzyme activity as a consequence of *t*-BuOOH treatment. On the other hand, dihydroferulic acid exhibited only a minor protective effects against cell cytotoxicity, lipid oxidation and glutathione depletion [115]. Since dihydrocaffeic acid is one of the main metabolites of yerba mate, its protective effect against oxidative liver damage may clarify the beneficial health effects, such as antioxidant and hepatoprotective properties associated with mate intake. The antioxidant activity of dihydrocaffeic acid was also confirmed in human EA.hy926 endothelial cells [116]. It was suggested that its protective effects resulted from the scavenging ability of intracellular ROS. Furthermore, dihydrocaffeic acid also enhanced nitric oxide synthase activity in a dose-related manner. Similarly, in a study from Wang et al., dihydrocaffeic acid prevented oxidative stress evoked by TNF-α and endothelial dysfunction in EA.hy926 cells [117].

The inhibition of lipid peroxidation by caffeic acid and 3,4-dihydroxyphenylacetic acid, a colonic metabolite of caffeic acid via dihydrocaffeic acid [67], was investigated in rat plasma [130]. In this study, these two acids suppressed the production of conjugated diene hydroperoxides and alpha-tocopherol consumption during the oxidation of soybean phosphatidylcholine multilamellar vesicles demonstrating their antioxidant properties in rat plasma, which represents a medium resembling the conditions in vivo. Four phenolic acids, namely 3-(3-hydroxyphenyl)propionic acid, 3,4-dihydroxyphenylacetic acid, 3-hydroxyphenylacetic acid, and 3-hydroxybenzoic acid, formed via colonic microbiota metabolism [67] in the human body after chocolate intake and excreted in urine, are known reducing agents contributing to antioxidant protective effects [139]. Colonic metabolite *m*-coumaric acid also exhibits potent antioxidant activity, while the antioxidant potency of microbial metabolite 3-(3-hydroxyphenyl)propionic acid was considerably lower compared to other phenolic acids in the oxygen radical absorbance capacity (ORAC) assay [132]. The antioxidant activity of hydroxybenzoic acids [140] and 4-hydroxyphenylacetic acid [134] were also confirmed in in vitro assays.

It has been proposed that polyphenolic compounds might also possess the indirect antioxidant activity via the upregulation of endogenous antioxidant enzymes in vivo [141]. In the in vivo study in rats, HCAs considerably enhanced the activity of antioxidant enzymes (i.e., NAD(P)H: quinone oxidoreductase 1, glutathione S-transferase, catalase, and glutathione peroxidase) indicating their chemoprotective properties [142]. Similarly, the activity of superoxide dismutase, catalase and/or glutathione peroxidase was increased following the administration of ferulic acid [143], the liposomal formulation of chlorogenic acid [144] or a single dose of coffee [145]. Due to the lack of information about indirect antioxidant activities of HCAs and their metabolites, there is a need for additional studies (especially in humans) in the future. 

### 4.2. Antimicrobial Activity

It has been known for many years that cinnamic acid and their derivatives possess antimicrobial activities. Antibacterial, antiviral and antifungal activities have been discovered for several natural and synthetic cinnamic acid derivatives, including HCAs [92]. However, in contrast to antioxidant activity, the aromatic hydroxyl group is not so crucial for antibacterial activity. In case of sinapic acid, the antibacterial activity has been confirmed in different studies on both plant and human pathogens [146]. In addition to inhibition of growth of some Gram-positive and Gram-negative bacteria, coumaric, ferulic and sinapic acids also showed some weak antifungal activity [92,147]. Rosmarinic acid is one of the HCAs that possess antiviral activity against many viruses (i.e., Herpes simplex, HIV, Japanese encephalitis virus) besides its antioxidant, antibacterial and antifungal properties [92]. Furthermore, L-chicoric acid and 3,5-dicaffeoylquinic acid were able to inhibit HIV-1 integrase and HIV-1 replication in tissue culture [148]. Regarding the antifungal activity, HCAs are toxic towards many fungal pathogens, including *Fusarium* species [149]. 

On the other hand, there have not been many studies describing the antimicrobial properties of HCAs’ metabolites (Table 1) up to date. Due to commercial unavailability, Heleno and coauthors synthesized protected forms of cinnamic and *p*-coumaric glucuronides and methylated metabolites to compare their antimicrobial activities with parent HCAs against many Gram-positive (*S. aureus*, *B. cereus*, *L. monocytogenes*, *M. flavus*), and Gram-negative bacteria (*P. aeruginosa*, *E. coli*, *S. typhimurium*, *E. cloacae*), and fungi [63,150,151]. Concerning the antibacterial activity, only the glucuronide protected form of *p*-coumaric acid retained antibacterial activity of the parent acid, whereas 2,3,4-tri-O-acetyl-1-cinnamoyl-D-glucuronic acid methyl ester showed lower activity than the parent compound. On the other hand, the measurement of antifungal activity disclosed higher potency compared to the respective parent HCAs against the majority of the evaluated fungi. Similarly, methylated derivatives of *p*-coumaric acid exhibited mostly higher antibacterial and antifungal activities than *p*-coumaric acid itself [63,150].

Keman and Soyer tested the ability of methicillin-resistant and susceptible *S. aureus* to attain the resistance against two phenolic compounds (2-hydroxycinnamic and vanillic acid) when being exposed to the subinhibitory concentrations [125]. The minimum inhibitory concentrations (MICs) obtained were 1.6 and 2.5 mg/mL for 2-hydroxycinnamic and vanillic acid, respectively. Furthermore, resistance to these acids could not be elicited, which shows their potential use as effective antimicrobials, especially in case of resistant pathogenic bacteria against antimicrobial agents [125]. 

Chlorogenic acids are the most abundant phenolic compounds from coffee being metabolized to hippuric acid in the human body [152]. The urine concentrations of hippuric acid ranging from 0.02 to 0.04 M are bacteriostatic at pH 5.0 for the prevailing pathogens of the urinary tract; however, only occasionally these concentrations could be achieved by cranberry juice [137]. It was shown that hippuric acid (2 mg/mL) possesses only limited antimicrobial activity at acidic pH values and has been used in combination with methenamine as urinary antiseptic for the prevention of recurrent urinary tract infections [153,154].

### 4.3. Anticancer Activity

Cinnamic acid has been of great interest among the scientific community due to its antioxidant, antiproliferative, antiangiogenic and antitumor activity [155]. Thus, numerous cinnamic acid derivatives have been assayed for their antitumor efficacy. Biological evaluation of numerous cinnamoyl acids, esters, amides, hydrazides and related derivatives in anticancer research was thoroughly reviewed by De, Baltas and Bedos-Belval in 2011 [156]. Furthermore, the anticancer potency of cinnamic acid derivatives was also summarized in a review by Su et al. in 2015 [155]. All major HCAs such as caffeic, ferulic and coumaric acids have shown anticancer properties in several studies, for example, in colon cancer [157], adenocarcinoma [158], hepatocarcinoma [159], breast cancer [160] and many other cancers. Similarly, the consumption of chlorogenic acids as one of the main phenolic component of coffee is also related to a reduced risk of several chronic diseases, including cancer [158,161,162]. The potential effects (including anti-cancerogenic) of chlorogenic acids on health have been summoned in a comprehensive review from Tajik et al. [11]. 

On the other hand, there have not been many reports about the anticancer properties of HCAs’ metabolites (Table 1). In general, the efficacy of phenolic acids and their metabolites varies from one compound to another due to structural diversity as well as variations in their molecular targets [163]. The presence of an aromatic ring and OH groups was identified as the key feature required for anticancer effects of phenolic compounds. Moreover, the potency increases with the number of hydroxyl groups compared to compounds lacking them or with methoxy substitution [163,164]. The study from Lee et al. implicates that *ortho* bis-hydroxylation and a tethered conjugated double bond are required for significant inhibitory potency on the growth of buccal mucosal and oral submucosus fibroblasts, neckmetastasis of Gingiva carcinoma and tongue squamous cell carcinoma cells [164]. Accordingly, HCA metabolites with aforementioned structural features could possess anticancer properties; however, many in vitro and preferably in vivo studies are required to confirm their potential anticancer activity, which has already been observed (and summarized in a review by Anantharaju et al. [163]) for their parent compounds. 

Nevertheless, a few studies of some HCA metabolites could be found in the literature (Table 1) [63,118,165]. Recently, it was reported that dihydrocaffeic acid, known as a metabolite of 5-O-caffeoylquinic and caffeic acids, was considerably more cytotoxic for tested cancer cell lines, i.e., breast adenocarcinoma (MCF-7), human prostate cancer cell line (PC-3), and colon carcinoma (HCT-116), compared to normal human cell line HDFa [118].

The cytotoxicity of *Coprinopsis atramentaria* methanolic extract along with *p*-hydroxybenzoic, *p*-coumaric and cinnamic acids, their synthetically prepared methylated and protected glucuronide derivatives was evaluated on five human cancer cell lines, namely MCF-7, HCT15, NCI-H460 (non-small cell lung carcinoma), HeLa (cervical carcinoma) and HepG2 (hepatocellular carcinoma) [165]. In most cases, methylated and glucuronated derivatives exhibited higher potencies in comparison with the corresponding parental compounds. The protected metabolites of cinnamic and *p*-coumaric acid, 2,3,4-tri-O-acetyl-1-cinnamoyl-D-glucuronic acid methyl ester (CAGP) and 2,3,4-tri-O-acetyl-1-*p*-coumaroyl-D-glucuronic acid methyl ester (CoAGP) respectively, showed higher cytotoxicity to cancer cells when compared to the corresponding parental acids [165]. 

The inducible enzyme cyclooxygenase-2 (COX-2) has an important role in regulating inflammation and potentially also in the development of colon cancer since several studies reported increased prostaglandin formation and COX-2 overexpression in human colon and colorectal cancer [136,166,167]. Two phenolic compounds (3-hydroxyphenylacetic acid and 3-(4-hydroxyphenyl)propionic acid) detected in the fecal water along with 3-phenylpropionic acid decreased the COX-2 levels in colonic HT-29 cells, which over time could be noteworthy for the prevention of tumor development in the colon [136]. 

Since ferulic acid could be metabolized into vanillic acid [168] and the anticancer activity of this metabolite might be important in an HCA- (chlorogenic, caffeic and especially ferulic acid) rich diet. It was reported that vanillic acid is an effective anticancer agent in vitro since at concentration of 0.6 mg/ml effectively induced oxidative stress and increased apoptosis in NCI-H460 cell line [126]. Furthermore, a noteworthy correlation was discovered between the content of vanillic acid, which is one of major phenolic acid in honeys, and inhibitory effects on breast and prostate cancer cell viability, proposing that vanillic acid could be the key ingredient responsible for the observed anticancer activity [127].

It was suggested that HCAs may also play a key role in colon cancer prevention (Table 1). The impact of chosen GIT metabolites of chlorogenic and caffeic acids, namely 3,4-dihydroxyphenylacetic acid and 3-(3,4-dihydroxyphenyl)propionic acid, on modulation of enzymes implicated in detoxification and inflammation (i.e., glutathione S-transferase T2 (GSTT2) and COX-2) in human adenoma cell line LT97 was analyzed by Miene and coauthors [119]. The upregulation of GSTT2 and the downregulation of COX-2 was observed, which probably contributes to the chemopreventive potential of HCAs after metabolic transformation in the gut. 3,4-dihydroxyphenylacetic acid also possessed antiproliferative activity in prostate and colon cancer cells showing considerably more potent inhibition of colon carcinoma (HCT116 cancer cell line) in comparison with immortalized normal intestinal epithelial cells IEC6 [131].

### 4.4. Anti-Inflammatory Activity

It is well known for polyphenols to display anti-inflammatory activity in vitro and in vivo by targeting inflammatory mediators, namely numerous cytokines (tumor necrosis factor α (TNF- α), interleukins (IL)), leukotrienes, and different enzymes (cyclooxygenases (COXs), inducible nitric oxide synthase (iNOS)) [169]. For instance, chlorogenic, caffeic and ferulic acids showed potent antioxidant and anti-inflammatory activities through the down-regulation of the LPS-induced expression of iNOS or COX-2 in RAW 264.7 macrophages [170]. In the in vivo study of intraperitoneal administration of ferulic acid in the Balb/c mice upregulation of antioxidant protection and suppression of inflammatory responses via the inhibition of TLR-4 induced activation of nuclear factor kappa B (NF-κB) was observed, which indicates the potential use of ferulic acid for protection against sepsis-induced acute kidney injury [171]. *p*-Coumaric acid, as one of the active ingredients in the Chinese natural herb *Oldenlandia difusa*, showed anti-inflammatory activity via the suppression of inflammatory cell infiltration as well as the levels of TNF-α and IL-6, which are known as pro-inflammatory factors that can activate the NF-κB signaling pathway, thereby increasing the inflammatory response and have an important role in the pathogenesis of rheumatoid arthritis [172]. Similarly, in another study *p*-coumaric acid exhibited considerable anti-inflammatory activity in vivo in arthritic rats via reduction of TNF-α expression in synovial tissue and circulating immune complexes in serum [173]. Recently, in a study in rat chondrocytes, *p*-coumaric acid reduced inflammatory responses caused by IL-1β via mitogen-activated protein kinase (MAPK) and NF-κB signaling pathway blockage; thus, it might be used to alleviate the symptoms of osteoarthritis [174]. Many other studies describing the anti-inflammatory activity of HCAs and synthetic derivatives were also reported [175,176]. Here, we will mainly focus on anti-inflammatory properties of HCAs’ metabolites (Table 1).

A study of six microbial metabolites (3,4-dihydroxyphenylpropionic, 3-(3-hydroxyphenyl)propionic, 3,4-dihydroxyphenylacetic, 3-hydroxyphenylacetic, 4-hydroxybenzoic and 4-hydroxyhippuric acids) using LPS-stimulated human PBMCs indicated that only dihydroxylated compounds, namely 3,4-dihydroxyphenylpropionic acid (dihydrocaffeic acid) and 3,4-dihydroxyphenylacetic acid, could provide the significant inhibition of pro-inflammatory cytokines (e.g., TNF-α, IL-1β and IL-6) secretion, which gives these compounds the potential to become a new class of therapeutic agents for the treatment of immuno-inflammatory diseases, such as atherosclerosis [120].

The in vitro anti-inflammatory effect via the determination of prostaglandin E2 production by IL-1β-stimulated colon fibroblast cells (CCD-18) was determined for 18 phenolic metabolites from gut microbiota [121]. The metabolites that significantly inhibited prostaglandin E2 production were ferulic, 3- and 4-hydroxyhippuric, 3,4-dihydroxyphenylacetic, dihydrocaffeic and dihydroferulic acids (the last three showed more than 50% inhibition). The latter exhibited a significant effect on PGE2 production inhibition even at a low dose (0.1 µM), which is close to the real levels of dihydroferulic acid in human plasma after the ingestion of cooked artichoke [121,177]. Furthermore, after intra-peritoneal administration in male Swiss albino mice at a dose of 30 mg/kg, 3,4-dihydroxyphenylacetic, dihydrocaffeic and dihydroferulic acids decreased the number of abdominal constrictions and enhanced the weight tolerance in the paw-pressure test, which demonstrates their systemic anti-inflammatory activity. Dihydroferulic acid inhibited lipid peroxidation and DNA damage in colon mucosa after the subcutaneous administration of carrageenan (to induce colitis) and diminished the expression of common inflammatory cytokines, namely TNF-α, IL-1β and IL-8 [121]. According to this study, it was suggested that a diet rich in dihydroferulic acid precursors (i.e., artichoke, cocoa, apples, strawberries, etc.) could employ anti-inflammatory effects and attenuate intestinal inflammation in humans.

The study from Kim et al. demonstrated anti-inflammatory effects for gut microbial metabolite vanillic acid (Table 1), which was able to inhibit LPS-induced TNF-α and IL-6 production, and suppressed the elevated levels of COX-2, prostaglandin E(2) and NO formation in mouse peritoneal macrophages and the activation of NF-κB and caspase-1 [128]. In another study, vanillic acid reduced the cluster of differentiation 40 ligand (CD40L)-induced vascular cell adhesion molecule-1 (VCAM-1) production in a linear dose-related manner [129]. CD40L as a ligand triggers the activation of CD40, which is connected to chronic inflammation promotion and expression of inflammatory mediators (e.g., VCAM-1 and IL-6). Vanillic acid via the suppression of VCAM-1 and IL-6 production shows the potential to modulate the progression of cardiovascular disease [129]. 

### 4.5. Neuro-, Cardio- and Hepato-Protective Effects

Due to the antioxidant activity, (poly)phenols and their metabolites exhibit protective effects on different tissues and organs in a human body. Neuro-, cardio- and hepato-protective effects have been acknowledged to HCAs and their metabolites (Table 1). Two HCAs’ metabolites 3,4-dihydroxyphenylpropionic acid (dihydrocaffeic acid) and 3,4-dihydroxyphenylacetic acid showed neuro-protective effects by preventing apoptosis of neurons by decreasing the ROS levels, enhancing redox activity, and decreasing oxidative stress-elicited apoptosis in human neuroblastoma SH-SY5Y cells [122]. According to results obtained in this study, it was suggested that a polyphenol-rich diet, which leads to metabolites such as 3,4-dihydroxyphenylpropionic and 3,4-dihydroxyphenylacetic acids (i.e., cocoa, tea, strawberry, walnut, and pomegranate), might lessen the oxidative stress related to the onset of neurodegenerative diseases; however, the detailed molecular mechanisms that eventually lead to the neuro-protective effects have not been elucidated up to date, so further studies are needed to confirm the beneficial effects of polyphenol-derived metabolites in vivo. 

A focal cerebral ischemia rat model was used to evaluate the neuro-protective effects of the metabolites of chlorogenic acids, focusing on dihydrocaffeic acid [123]. It was shown that dihydrocaffeic acid dose dependently decreased brain infarct volume, behavioral deficits, brain water content, and Evans Blue leakage in rats. The inhibitory effects of dihydrocaffeic acid on blood–brain barrier damage might emerge due to the inhibitory effect on matrix metalloproteinase MMP-2 and -9 expressions and activities [123]. Thus, dihydrocaffeic acid might represent the major active metabolite responsible for the protective effects of chlorogenic acids in case of ischemic stroke.

The metabolites, which can be connected to coffee-derived chlorogenic acid intake, namely dihydrocaffeic acid, dihydroferulic acid and feruloylglycine, increased the vitality of the human neuroblastoma SK-N-MC cells by 16% higher protection compared to untreated cells [178].

The cardio-protective effects of HCAs and their metabolites were described in a study by Baeza et al. [124]. Caffeic and ferulic acids and their GIT metabolites dihydrocaffeic and dihydroferulic acids decreased the ADP-induced P-selectin expression that triggers the inhibition of excessive platelet activation, which could lead to the physical blocking of blood vessels and chronic inflammation, both recognized as independent risk factors for cardiovascular disease. Dihydrocaffeic and dihydroferulic acids were found as more potent inhibitors of P-selectin expression in comparison with their parental phenolic precursors [124].

The evaluation of hepato-protective effects of dihydrocaffeic acid in HepG2 cells, which were exposed to oxidative damage elicited by *t*-BuOOH showed that dihydrocaffeic acid was able to prevent the cytotoxicity and macromolecular damage along with the previously mentioned antioxidant activity [115].

Colonic metabolite of HCAs 4-hydroxyphenylacetic acid was able to prevent acute liver injury in mice evoked by acetaminophen via inhibitory effects on CYP2E1 and the up-regulation of phase II and antioxidant enzymes as a result of Nrf2 activation [135].

### 4.6. Other Activities

In addition to their antioxidant, antimicrobial, anticancer and anti-inflammatory activities, HCAs and their derivatives also exhibit anti-collagenase and anti-tyrosinase activities in addition to UV-protective effects; therefore, they might be employed as preservatives, anti-aging, anti-inflammatory, and hyperpigmentation-correcting agents [90]. Some activities were also found for HCAs’ metabolites. For example, the protection of keratinocytes from UV irradiation by suppressing the expression of IL-6 and IL-8 was demonstrated for dihydrocaffeic acid [179]. HCAs also showed antidepressant activity. Ferulic acid is the most studied HCA, which was able to raise the monoamine neurotransmitter levels in the brain [180] and neutralize the reduction in reward-seeking behavior [181]. Other cinnamic acids (e.g., 3,4,5-trimethoxycinnamic, caffeic and *p*-coumaric acids) were also investigated for their potential antidepressant effects (all studies are reviewed in a recent paper by Diniz et al. [181]).

Antihypertensive effects were also discovered for one of the main metabolites of ferulic acid, namely ferulic acid-4-O-sulfate (Table 1) [114]. In a study where the comparison of ex vivo vasorelaxing effect of ferulic acid and ferulic acid-4-O-sulfate on isolated mouse arteries mounted in tissue myographs was performed, only the latter exhibited the dose-dependent vasorelaxation of saphenous and femoral arteries and aortae. Therefore, it was suggested that ferulic acid-4-O-sulfate is most likely one of the major metabolites involved in the antihypertensive effect related to the consumption of ferulic acid [114]. Another HCAs’ gut microbiota metabolite 3-(3-hydroxyphenyl)propionic acid, which is also one of the metabolites of flavonoid quercetin, showed more potent antihypertensive effects in vivo compared to their parent compound in both healthy and spontaneously hypertensive rats. The proposed mechanism of action involves NO-dependent effects and peripheral activity on vascular beds [133]. The inhibitory activity of caffeic acid derivatives on key enzymes associated with hypertension (angiotensin-converting enzyme [107,182], ectonucleoside triphosphate diphosphohydrolase, 5ʹ-ectonucleotidase, adenosine deaminase, acetylcholinesterase, and arginase [107]) were also described recently.

Furthermore, it is well known that HCAs and their derivatives also show promising antidiabetic properties [55,183,184]. In vitro inhibitory effects of *Echinacea purpurea* flower extract and selected caffeic acid derivatives on key enzymes important in the management of type 2 diabetes mellitus (i.e., α-amylase and α-glucosidase) [182] were published recently. Even though many mechanisms for the involvement of HCAs in the prevention and management of diabetes and its complications have been proposed, there is still a lack of information about the potential antidiabetic activity of their metabolites and no clinical evidence to prove their beneficial effects [184]. 

## 5. Conclusions

Hydroxycinnamic acids (HCAs) represent one of the most important classes of natural (poly)phenolic compounds, being present in high concentrations in fruits, vegetables, cereals, and drinks (tea, coffee, wine). A range of health beneficial effects were observed for HCAs and in recent years, also for their GIT, liver and kidneys’ metabolites. The most common metabolic reactions convert the HCAs into more polar glucuronides and sulfates as well as methylated derivatives, which either retain, enhance or lose the biological activity of corresponding parental HCAs. Furthermore, HCAs’ metabolism by gut microbiota involves many other biochemical transformations (β-oxidations, (de)hydrogenations, (de)methylations, dihydroxylations, etc.), leading to diverse metabolites in human plasma and urine following the HCAs-rich diet. Since this diet has been associated to several beneficial health effects, HCAs and their metabolites can have important roles in various biological pathways in the human body. Their health effects rely on their concentration in plasma and tissue compartments; however, only small amounts of free forms of ingested HCAs are usually present there. On the other hand, structurally diverse metabolites can reach higher concentrations, thus being responsible for the potential beneficial health effects of parental HCAs. Even though many biological activities have been acknowledged to HCAs’ metabolites in vitro, there is still a lack of in vivo studies. Nevertheless, HCAs and also a few of their metabolites are known for their potent direct or also indirect antioxidant activity, thereby being important for the prevention or potential treatment of oxidative stress-related diseases, such as neurodegenerative and cardiovascular diseases, cancer and various inflammatory conditions. Neuro-, cardio- and hepato-protective effects have been described for HCAs and their metabolites and many studies on different cancer cell lines also showed their potential intervention in cancer diseases. However, the data obtained in in vivo studies in animal models and human are still scarce and inconsistent. Additional epidemiological and clinical studies are needed to demonstrate the beneficial health effect of HCAs and elucidate the underlying mechanism of action. And finally, potential negative effects of HCA and their metabolites on human health have to be taken into account, even though the majority of HCAs are considered safe, especially at the concentrations present in the human plasma and tissue compartments. Currently, there is still no or very little information concerning their safety, especially in terms of metabolic transformations that occur in the human body.

## Figures and Tables

**Table 1:** Biological activities and underlying mechanism of major HCAs’ metabolites.

Metabolite(s)	Biological Activity	Mechanism	Reference(s)
3-(3,4-dihydroxyphenyl)lactic acid	antioxidant	radical scavenging ability (in vitro DPPH assay)	[88]
caffeic acid-3-O- and 4-O-sulfate	antioxidant	ferric-reducing activity, reduction of the ABTS radical cation (in vitro assays)	[112]
caffeic acid-3-O- and 4-O-glucuronide	antioxidant	ferric-reducing activity, reduction of the ABTS radical cation (in vitro assays)	[112]
ferulic acid-4-O-glucuronide	antioxidant	potent activity in CuSO_4_-induced LDL autoxidation system (in vitro assay)	[113]
caffeic acid-4-O-sulfate	antihipertensive	vasorelaxation of saphenous and femoral arteries and aortae in mice	[114]
ferulic acid 1-O-acyl-glucuronide	antioxidant	ferric-reducing activity, reduction of the ABTS radical cation (in vitro assays)	[112]
dihydrocaffeic acid	antioxidant	DPPH and lipid peroxyl radical scavenging ability,	[111]
		increased oxidative stability of lard (in vitro assays)
		dose-dependent recovery of reduced glutathione and increased ROS levels in HepG2 cells,	[115]
		scavenging of intracellular ROS species in endothelial cells,	[116]
		enhanced nitric oxide synthase activity in a dose-related manner in EA.hy926 cells
		prevention of oxidative stress and endothelial dysfunction in EA.hy926 cells	[117]
	anticancer	cytotoxicity for tested cancer cell lines (i.e., MCF-7, PC-3, and HCT-116)	[118]
		chemoprotective - upregulation of GSTT2 and downregulation of COX-2 in human colon cells (LT97)	[119]
	anti-inflammatory	decreased secretion of pro-inflammatory cytokines TNF-α, IL-1β and IL-6 in human	[120]
		prostaglandin E2 production inhibition in vitro in CD18-Co human colon fibroblast cells	[121]
		reduced number of abdominal constrictions,	
		higher weight tolerance in the paw-pressure test in rats	
	neuroprotective	prevention of neuronal apoptosis by reducing the ROS levels, enhanced redox activity, and reduced oxidative stress-elicited apoptosis in human neuroblastoma SH-SY5Y cells;	[122]
		dose-dependent reduction of brain infarct volume, behavioral deficits, brain water content, and Evans Blue leakage in focal cerebral ischemia rat model;	[123]
		inhibition of expression and activation of MMP-2 and -9;	
	cardio-protective	Inhibition of in vitro platetet activation via decreased P-selectin expression	[124]
	hepato-protective	prevention of cytotoxicity, macromolecular damage in *t*-BuOOH-challenged HepG2 cells	[115]
dihydroferulic acid	antioxidant and hepatoprotective	minor protective effects against cell cytotoxicity, lipid oxidation and glutathione depletion in HepG2 cells	[115]
	anti-inflammatory	prostaglandin E2 production inhibition in vitro in CD18-Co human colon fibroblast cells, reduced number of abdominal constrictions, enhanced weight tolerance in the paw-pressure test in rats;	[121]
		inhibition of DSS-induced colitis, lipid peroxidation and DNA damage in colon mucosa,	
		downregulation of central pro-inflammatory cytokines (e.g., TNF-α, IL-1β, and IL-8)	
	cardio-protective	inhibition of in vitro platetet activation via decreased P-selectin expression	[124]
vanillic acid	antibacterial	inhibition of growth of methicillin-resistant and methicillin-susceptible *S. aureus*	[125]
	anticancer	increased oxidative stress and apoptosis in non-small lung cancer NCI-H460 cell line	[126]
		inhibition of breast and prostate cancer cell viability	[127]
	anti-inflammatory	reduced LPS-induced production of TNF-α and IL-6,	[128]
		suppression of the elevated levels of COX-2, production of prostaglandin E(2) and NO in mouse peritoneal macrophages and activation of NF-κB and caspase-1;	
		reduction of CD40L-induced production of VCAM-1 production suppression of IL-6 production in oxidatively challenged HUVECs	[129]
3,4-dihydroxyphenylacetic acid	antioxidant	inhibition of lipid peroxidation in rat plasma	[130]
	anticancer	chemoprotective - upregulation of GSTT2 and downregulation of COX-2 in human colon cells (LT97)	[119]
		antiproliferative activity in prostate and colon cancer cells	[131]
	anti-inflammatory	decreased secretion of pro-inflammatory cytokines TNF-α, IL-1 β and IL-6 in human;	[120]
		blockage of prostaglandin E2 production in vitro in CD18-Co human colon fibroblast cells	[121]
	neuroprotective	prevention of neuronal apoptosis by reducing the ROS levels, enhanced redox activity, and reduced oxidative stress-elicited apoptosis in human neuroblastoma SH-SY5Y cells;	[122]
3-(3-hydroxyphenyl)propionic acid	antioxidant	antioxidant activity in ORAC assay (in vitro assay)	[132]
	antihypertensive	NO-dependent effects, peripheral activity on vascular beds in rats	[133]
4-hydroxyphenylacetic acid	antioxidant	radical scavenging ability in in vitro DPPH assay	[134]
	hepato-protective	up-regulation of phase II and antioxidant enzymes via Nrf2 activation in mice	[135]
3-hydroxyphenylacetic and 3-(4-hydroxyphenyl)propionic acids	anti-inflammatory and anticancer	decrease in the COX-2 levels in colonic HT-29 cells	[136]
3- and 4-hydroxyhippuric acid	anti-inflammatory	blockage of prostaglandin E2 production in vitro in CD18-Co human colon fibroblast cells	[121]
hippuric acid	antimicrobial	bacteriostatic for the common pathogens in the urinary tract	[137]
